# Unsupervised learning for prognostic validity in patients with chronic pain in transdisciplinary pain care

**DOI:** 10.1038/s41598-023-34611-z

**Published:** 2023-05-10

**Authors:** Irina A. Strigo, Alan N. Simmons, Jameson Giebler, Jan M. Schilling, Tobias Moeller-Bertram

**Affiliations:** 1 Emotion and Pain Laboratory, San Francisco Veterans Affairs Health Care Center, 4150 Clement Street, San Francisco, CA 94121 USA; 2 Department of Psychiatry, University of California San Francisco, 401 Parnassus Ave, San Francisco, CA 94143 USA; 3 Stress and Neuroimaging Laboratory, San Diego Veterans Affairs Health Care Center, 3350 La Jolla Village Drive, MC 151-B, San Diego, 92151 USA; 4 Department of Psychiatry, University of California San Diego, 9500 Gilman Dr., La Jolla, CA 92093 USA; 5 Center of Excellence in Stress and Mental Health, San Diego Veterans Affairs Health Care Center, 3350 La Jolla Village Dr., San Diego, CA 92161 USA; 6 Savas Health, Rancho Mirage, CA USA; 7 Vitamed Research, LLC, Palm Desert, CA USA

**Keywords:** Comorbidities, Pain, Outcomes research, Predictive markers, Prognostic markers

## Abstract

Chronic pain is not a singular disorder and presents in various forms and phenotypes. Here we show data from a cohort of patients seeking treatment in a transdisciplinary pain clinic. Patients completed a multidimensional patient-reported battery as part of routine initial evaluation at baseline and at each of the four subsequent visits over 1-year follow-up (0, 1, 3, 6, 12 months). The goal of this work was to use unsupervised modeling approach to identify whether patients with chronic pain undergoing transdisciplinary intensive rehabilitation treatment: (1) can be derived based upon self-reported outcome measures at baseline (or before treatment initiation), (2) are clinically validated based on their clinical diagnosis and medication use, and (3) differ in treatment trajectories over 1 year of transdisciplinary treatment. We applied unsupervised clustering on baseline outcomes using nine patient-reported symptoms and examined treatment trajectories. The three-cluster solution was internally validated. Psychiatric diagnosis, chronic back pain-related disability and symptoms severity determined cluster assignment and treatment prognosis. Conversely, clinical pain severity had lesser effect. Furthermore, clusters showed stability over time despite symptoms improvement. The accurate and meaningful subgrouping of the underlying chronic pain phenotypes would greatly enhance treatment and provide personalized and effective pain management.

## Introduction

Chronic pain affects up to 60 million people worldwide and the annual cost of chronic pain in the United Sates is as high as $635 billion a year, more than the yearly costs for cancer, heart disease, and diabetes, combined. Chronic pain is not a singular disorder^1^. Beyond the diversity of its representation, the comorbidities associated with chronic pain lead to further heterogeneity^2^, treatment failures or marginal treatment results^3^. The accurate and meaningful subgrouping of the underlying pain phenotypes among patients with chronic pain would greatly enhance treatment strategies and effectiveness^4^. Data-driven approaches that characterize patients with similar diagnosis based on patient-reported outcomes show great promise in understanding clinical conditions^5,6^. Clustering approaches have been informative in the detection of subsamples among chronic pain patients^4,7–9^ and, potentially, in predicting patients’ prognosis^4^. A recent study in a large sample of real-world patients seeking treatment at a tertiary academic pain clinic found three robust clusters in their sample defined by graded symptoms severity with negative-affect-related symptoms being a key determinant in cluster separation^4^. Symptom severity at follow-up was predicted by baseline cluster assignment in this study suggesting cluster stability and, potentially, trait-based than state-based cluster characterization^4^. Although informative, translating these classification systems into effective treatments approaches has been challenging, as longitudinal data from specific and controlled treatment approaches are lacking.

The current study examined the trajectories of treatment effects of the transdisciplinary treatment approach for patients with chronic pain through the completion and collection of study subject questionnaires (patient-reported outcomes) and applying unsupervised modeling. The subjects were assessed at different time points (pre-treatment and then at months 1, 3, 6, then again after 12 months). Here we wanted to investigate whether robust subgroups of subjects (1) can be derived based upon self-reported outcome measures at baseline, i.e., before initiation of treatment, (2) are clinically validated, and (3) differ in treatment trajectories over a year of follow-up treatment. We also examined whether the services used in the transdisciplinary pain care differed between the subgroups and explored cluster dynamics over the course of treatment. Our large longitudinal dataset with multiple assessments points builds on the existing models of the underlying chronic pain phenotypes^4^ and provides unique evidence for patients’ prognosis following transdisciplinary pain management. As transdisciplinary pain care is becoming a “go to” pain management approach^10^, we believe our findings will: (1) increase knowledge of the chronic pain illness experience and effects of treatment on patient’s physical and mental functioning, and (2) provide initial evidence for personalized treatment assignment and optimization.

## Results

### Cluster discovery

**Figure 1:**
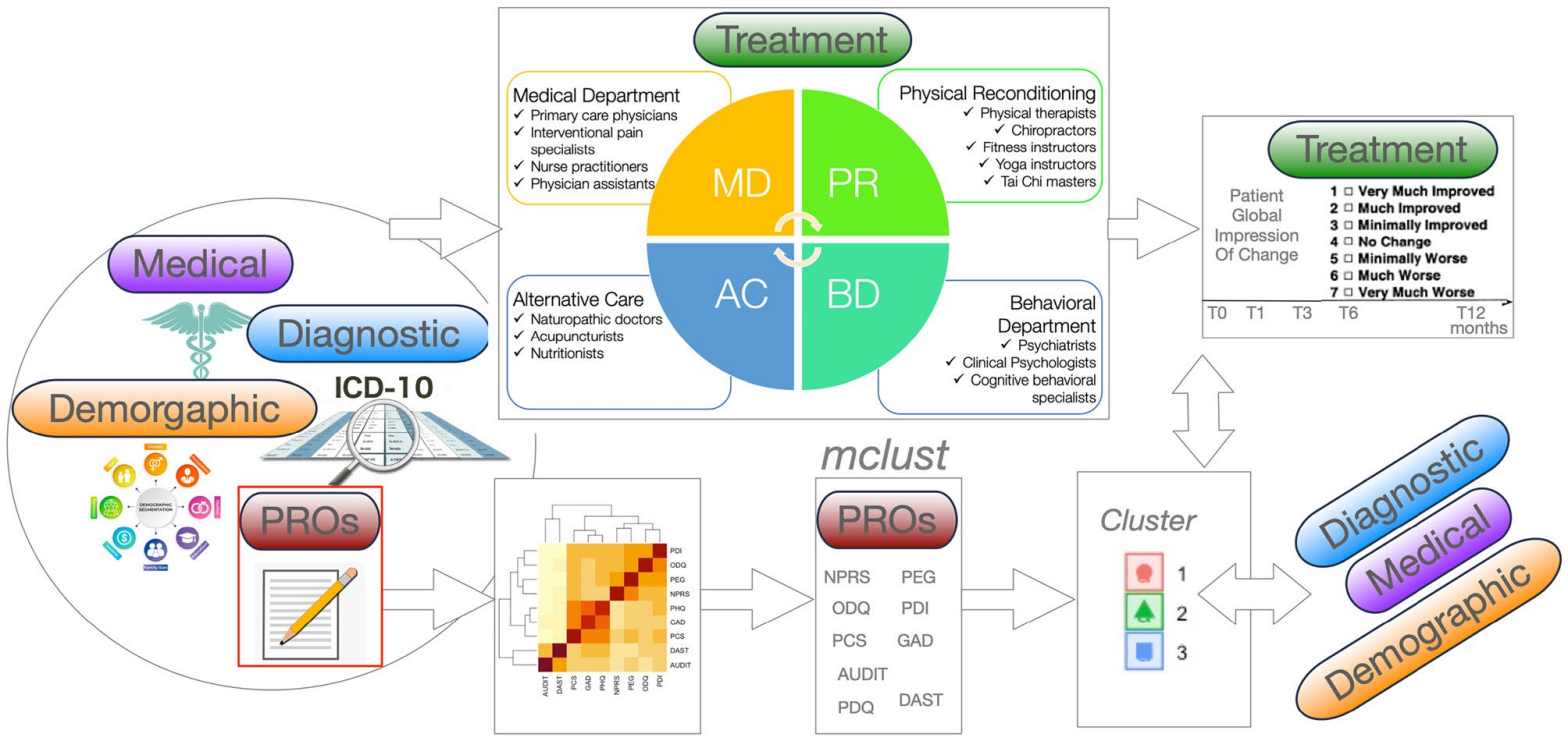
Study flow schematic. Demographic, medical, diagnostic and patient reported outcomes of pain disability, pain-related cognitions and psychological symptoms are collected before patients begin transdisciplinary pain treatment. The four departments of the care are shown (top, Medical Department (MD), Behavioral Department (BD), Physical Reconditioning Department (PR), and Department of Alternative Care (AC). Patient reported outcomes (PROs) were collected at baseline and modelled with unsupervised clustering approach (*Mclust* in *R,* c.f. text for details)*.* Treatment trajectories were examined with Patient Global Impression of Change (PGIC). Clusters were compared on treatment outcomes as well as baseline diagnostic, medical and demographic characteristics. (c.f. text for further details).

Study design is summarized in Fig. 1. A total of 3296 patients subjects included in the current analysis completed self-reported outcomes battery at baseline, or Time 0. Examination of data completion at baseline and at each of the following time points in the overall sample, indicated 69% completion at month 1, 56% at month 3, 40% at month 6 and 20% at month 12. Cluster analysis (*Mclust*) produced a three-group solution based on subjects’ baseline responses (Time 0), sorting 1064 patients into Cluster1 (MCL1), 1709 patients into Cluster2 (MCL2) and 523 patients into Cluster 3 (MCL3). Questionnaire completion was the same in each of the three cluster groups (χ^2^ = 0.64, p = 0.99), thus longitudinal analysis was conducted in completers to minimize inference.

### Cluster characterization: demographics

Mclust subgroups demographic characteristics are shown in Table 1. Several notable differences in demographic variables were observed. The subgroups differed significantly on age (F(2) = 5.781, p < 0.01). Post-hoc comparisons showed that MCL2 was slightly but significantly younger than MCL1 (p < 0.01, Holm corrected). No other comparisons were significant. Likewise, sex distribution was significantly different between the three cluster subgroups (χ^2^ = 69.129, p < 0.001). Whereas proportion of male/females was similar in MCL1 (36%/64%) and MCL2 (30%/70%), it differed in MCL3 (50%/50%).

**Table 1:** Subjects characteristics.

MClust group	MCL1		MCL2		MCL3		Stats
	Mean	SD	Mean	SD	Mean	SD	F, t, χ^2^/pval
N	1064		1709		523		
Age	51.531	12.113	49.964	11.973	50.364	10.934	5.781/0.003
Sex (M/F) [%]	385/679	%36/64	520/1189	%30/70	262/261	%50/50	68.151/< 0.001
Num visit	82.983	70.168	88.714	73.072	90.358	74.31	2.823/0.06
Days total	201.327	179.526	217.702	180.517	225.297	170.119	3.762/0.023
Blood pressure							
SBP (base)	131.965	45.935	128.48	18.907	133.062	59.651	2.496/0.089
DBP (base)	84.365	39.941	81.44	27.884	82.304	11.688	2.621/0.073
BMI (base)	32.345	8.806	33.008	8.766	30.553	7.869	8.857/< 0.001
MEDD	24.701	53.015	28.338	56.962	34.007	79.176	3.439/0.032
Diagnosis (ICD-10)	No	Yes	No	Yes	No	Yes	
M54	7.490%	92.510%	7.331%	92.669%	8.031%	91.969%	0.253/0.881
M99	15.714%	82.260%	15.977%	84.023%	16.252%	83.748%	0.086/0.958
M47	89.736%	10.264%	89.097%	10.903%	88.697%	11.303%	0.469/0.791
M25	52.896%	47.104%	57.049%	42.951%	58.700%	41.300%	7.725/0.021
M79	79.462%	20.538%	85.468%	15.132%	86.233%	13.767%	19.807/< 0.001
F33	66.472%	33.528%	71.992%	28.008%	57.361%	42.639%	34.032/< 0.001
G89	82.972%	17.028%	75.846%	24.154%	89.293%	10.707%	46.354/< 0.001
Medication							
Gabapentin	47.162%	52.838%	48.496%	51.504%	45.507%	54.493%	1.297/0.523
Hydrocodone.acetamin	49.036%	50.965%	46.043%	53.947%	49.140%	50.860%	2.611/0.271
Diclofenac	50.146%	49.854%	53.008%	46.992%	53.728%	46.272%	3.237/0.198
Lidocaine	57.461%	42.539%	60.996%	39.004%	58.126%	41.874%	3.468/0.177
Meloxicam	62.083%	37.917%	61.372%	38.628%	62.524%	37.476%	0.236/0.889
Baclofen	71.036%	28.964%	67.951%	32.049%	68.834%	31.166%	3.170/0.205
Ibuprofen	63.546%	36.454%	64.004%	35.996%	64.054%	35.946%	0.080/0.961
Omeprazole	72.616%	27.384%	75.282%	24.718%	75.717%	24.283%	3.408/0.182
Atorvastatin	77.531%	22.469%	75.658%	24.342%	77.247%	22.753%	1.341/0.512
Cyclobenzaprine	71.036%	28.964%	72.462%	27.538%	70.937%	29.063%	0.745/0.689

*SBP* systolic blood pressure, *DBP* diastolic blood pressure, *BMI* body mass index, *MEDD* morphine equivalent daily dose, *ICD-10* International Classification of Diseases.

### Cluster characterization: patient reported outcomes

**Figure 2:**
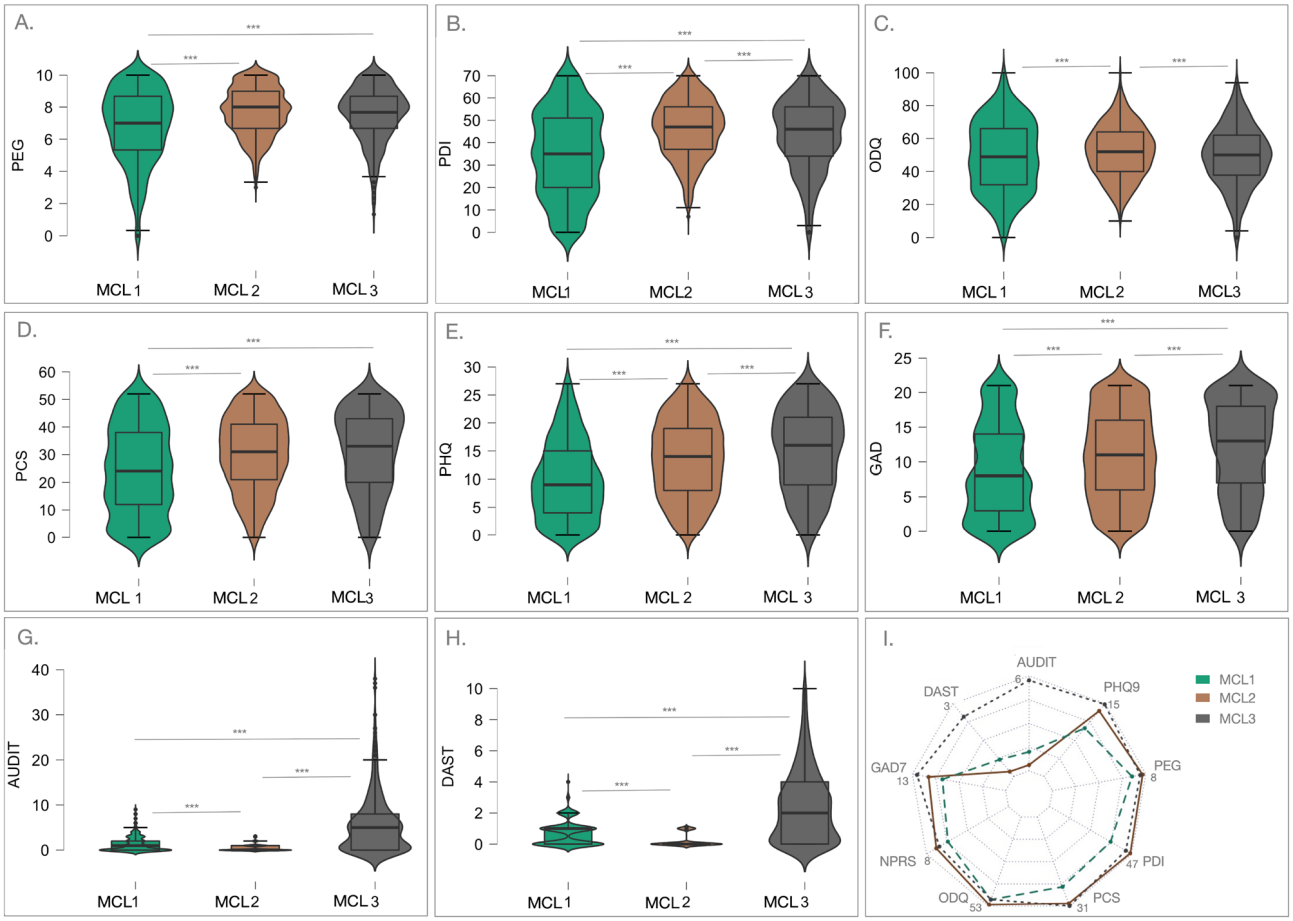
Violin plots of Patient Reported Outcomes at baseline. One-way ANCOVAs with Mclust subgroup as a fixed factor, sex and age as covariates showed significant subgroup for: (**A**) Pain impact (PEG; F(2, 1, 1) = 84.105, p < 0.001); (**B**) Pain-related disability (PDI; (F(2,1,1) = 174.338, p < 0.001); (**C**) back-pain related disability (ODQ) (F(2,1,1) = 12.253, p < 0.001); (**D**) Pain catastrophizing (PCS; F(2, 1,1) = 62.029, p < 0.001); (**E**) Depressive symptoms severity** (**PHQ9; F(2, 1, 1) = 119.547, P < 0.001); (**F**) Anxiety symptoms severity** (**GAD: F(2, 1, 1) = 59.784, P < 0.001); (**G**) Alcohol use test (AUDIT; F(2, 1,1) = 748.796, p < 0.001); and (**H**) Drug abuse screening test (DAST**;** (F(2, 1, 1) = 908.834, p < 0.001); (**I**) Graphic representation of patient reported outcomes among the three unsupervised Mclust subgroups. The radar plot is bounded by the minimum (0) and maximum scores in the current sample for each scale (these averages for each subgroup are displayed for reference). Subgroup 1 [MCL1, green, n = 1064], Subgroup 2 [MCL2, brwon, n = 1709], and Subgroup 3 [MCL3, grey, n = 523]. *AUDIT* The Alcohol Use Disorders Identification Test, *DAST* The Drug Abuse Screening Test, *PCS* pain catastrophizing scale, *PEG* pain impact, *PHQ* patient health questionnaire 9 (depressive symptoms), *GAD* general anxiety disorder symptoms, *NPRS* numerical pain rating scale, *ODQ* Oswestry disability questionnaire, *PDI* pain disability index. Post-hoc comparisons between subgroups are shown *p < 0.05, **p < 0.01. ***p < 0.005. c.f. text for further details.

Examination of baseline outcome measures showed that Mclust subgroups differed on several patient reported outcomes. This is graphically demonstrated by the radial plot in Fig. 2i. As can be seen in Fig. 2i, MCL1 can be described by low scores on all of the self-reported outcomes, except for substance use measures. Conversely, MCL2 can be described by high scores of pain, pain-related catastrophic thinking and disability, while MCL3, can be described by high scores on psychiatric measures, pain-related catastrophic thinking and disability (not due to back pain). Mclust subgroup detailed scores are shown in Fig. 2a–h. Examination of pain-related outcomes with the use of one-way ANCOVA with Mclust subgroup as a fixed factor, sex and age as covariates showed that PEG scores, although clinically very similar (i.e., all falling into moderate-severe range), were significantly different between the three subgroups (F(2, 1, 1) = 84.105, p < 0.001) (Fig. 2a). Post-hoc comparisons of the Mclust subgroups showed that MCL1 scored significantly less than MCL2 and MCL3 (p < 0.001, Holm corrected) and there was no significant difference between MCL2 and MCL3 (note p = 0.074, Holm corrected). NPRS scores showed similar results to that of PEG (F(2,1,1) = 133.744, p < 0.001). Post-hoc comparisons showed that all groups were significantly different with MCL1 scoring the lowest, MCL2 the highest and MCL3 scoring slightly (both groups providing an average of 7/10 on NPRS), but significantly, lower than MCL2 (p’s < 0.005, Holm corrected). Pain disability outcomes showed a slightly different relationship between the subgroups. One-way ANCOVAs with Mclust subgroup as a fixed factor showed significant effect for both PDI (F(2,1,1) = 174.338, p < 0.001) and ODQ (F(2,1,1) = 12.253, p < 0.001). However, post-hoc comparisons showed a slightly different relationship between the subgroups. For PDI, the relationship was similar to PEG and NPRS with MCL1 scoring the lowest, MCL2 the highest and MCL3 scoring slightly but significantly lower than MCL2 (p’s < 0.005, Holm corrected) (Fig. 2b). For ODQ, which is more specific to back pain-related disability, MCL2 scored the highest, which was significantly higher than that in MCL1 (p < 0.001) and MCL3 (p = 0.004) and there was no significant difference between MCL1 and MCL3 (p = 0.647, Holm corrected) (Fig. 2c). Examination of PCS scores also showed significant effect (F(2, 1,1) = 62.029, p < 0.001). Post-hoc comparisons showed that MCL1 scored the lowest, which was significantly lower than that in MCL2 and MCL3 (p’s < 0.001) and there was no significant difference between MCL2 and MCL3, although it approached significance (p = 0.056, Holm corrected) (Fig. 2d). Psychiatric variables showed slightly different relationship compared to pain-related outcomes and cognitions. As determined by one-way ANCOVA with Mclust subgroup as a fixed factor, and sex and age as covariates both, PHQ (Fig. 2e) and GAD (Fig. 2f) scores showed significant subgroup differences (PHQ9: F(2, 1, 1) = 119.547, P < 0.001; GAD: F(2, 1, 1) = 59.784, P < 0.001). Post-hoc comparisons were all significant on both PHQ9 and GAD measures with MCL1 scoring the lowest, MCL3 the highest and MCL2 scoring slightly but significantly less that MCL3 (p’s < 0.001, Holm corrected). Likewise, AUDIT scores were significantly different (F(2, 1,1) = 748.796, p < 0.001). Post-hoc comparisons of the Mclust subgroups showed that all three subgroups were significantly different with MCL2 scoring the lowest, MCL3 the highest and MCL1 scoring in between the other two groups (p’s < 0.001, Holm corrected) (Fig. 2g). Similar results were observed with the DAST scores (F(2, 1, 1) = 908.834, p < 0.001), showing the same significant relationship between the three subgroups with MCL2 scoring the lowest, MCL3 the highest and MCL1 scoring in between the other two groups (p’s < 0.001, Holm corrected) (Fig. 2h).

### Cluster characterization: clinical validation

To determine clinical validation of our subgroups, we examine the degree to which our baseline cluster analysis of patient reported outcomes agreed with the clinical presentation. All clinical characteristics of subgroups are shown in Table 1. There were no significant between subgroup differences in baseline blood pressure (SBP: F(2,1,1) = 2.496, p = 0.089; DBP: F(2,1,1) = 2.621, p = 0.073). There was a significant difference in the baseline body mass index (BMI) (F(2,1,1) = 8.857, p < 0.001). Post-hoc analysis showed that MCL3 had lower BMI at baseline than the other two subgroups (p’s < 0.01). There was no significant between group differences in the proportion of patients on opioid medication (χ^2^ = 3.847, p > 0.1) with ~ 80% of individuals taking opioids in each of the subgroups. Nevertheless, we found significant between subgroup difference in the amount of prescribed opioids (measured by Morphine Equivalent Daily Dose, MEDD) (F (2,1,1) = 3.439, p = 0.032). Post-hoc comparison showed that MCL3 were prescribed more opioid medication that MCL1 (p < 0.05). We found that severe back pain (ICD-10: M54) was the most prevalent diagnosis in all the three subgroups with the overall prevalence of ~ 92% (χ^2^ = 0.254, p = 0.881), followed by the biomechanical lesions (ICD-10: M99) with the overall prevalence of ~ 84% (χ^2^ = 0.086, p = 0.958). We found that other joint disorder, not elsewhere classified (ICD-10: M25) was most prevalent in MCL1, least in MCL3 and MCL2 falling in the middle (χ^2^ = 7.725, p = 0.021), with overall prevalence of ~ 45%. Likewise, soft tissue disorders/myalgia (ICD-10: M79), was most prevalent in MCL1, least in MCL3 and MCL2 falling in the middle (χ^2^ = 19.807, p < 0.0001), with the overall prevalence ~ 18%. Notably, fibromyalgia, (ICD-10: M79.7) was the most prevalent diagnosis under this category (45%). Pain not elsewhere classified (ICD-10: G89), was most prevalent in MCL2, least in MCL3 and MCL1 falling in the middle (χ^2^ = 46.354, p < 0.0001), with overall prevalence ~ 18%. Recurrent depressive disorder (ICD-10: F33) was most prevalent in MCL3, least in MCL1 and MCL2 falling in the middle (χ^2^ = 34.032, p < 0.00001), with the overall prevalence ~ 33%. Examination of most commonly prescribed medications in our study did not show significant differences between the three subgroups (see Table 1).

### Cluster characterization: resource utilization

**Figure 3:**
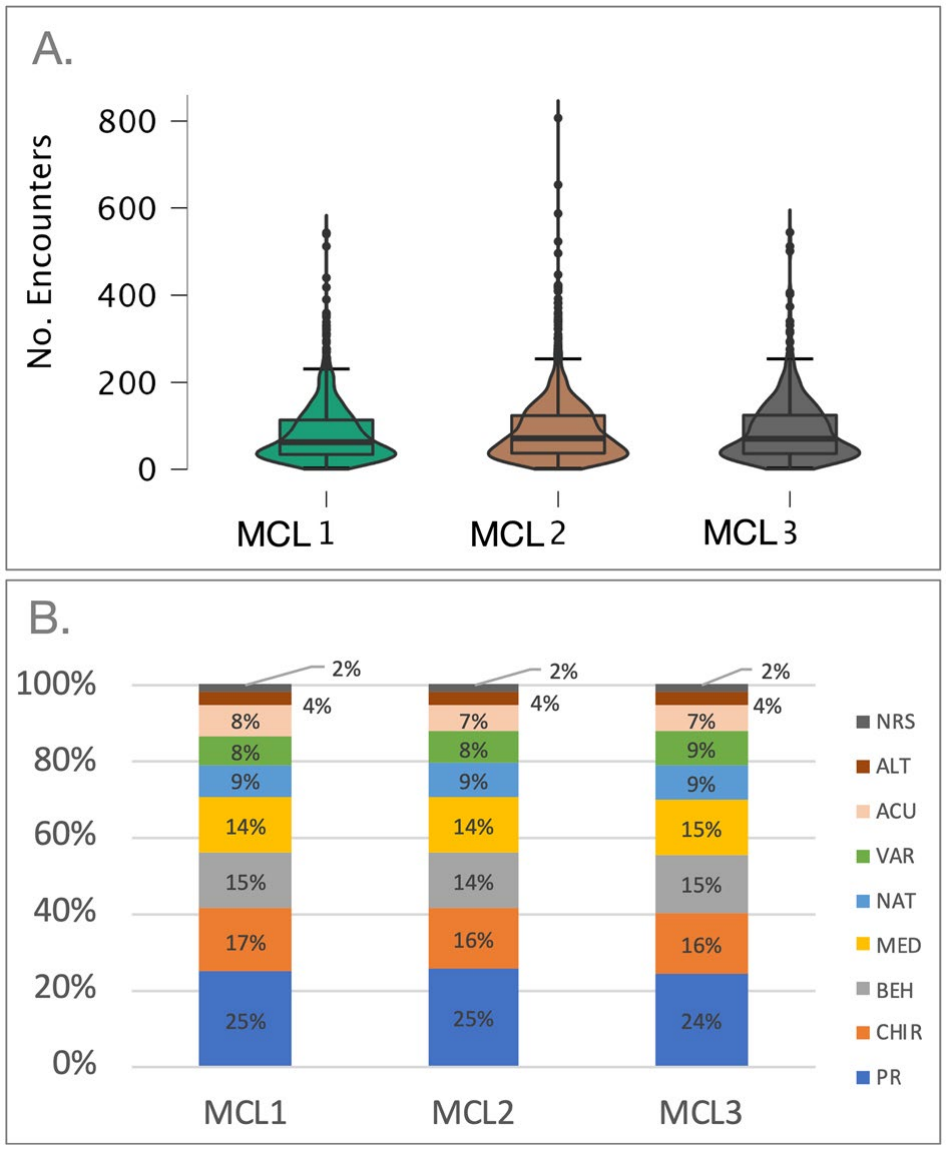
Patient encounters with medical providers and services. (**A**) Total number of encounters per Mclust subgroup. One-way ANCOVA with Mclust subgroup as a fixed factor, sex and age as covariates showed that groups approached significance in the number of visits/encounters (F(2,1,1) = 2.823, p = 0.060). Post-hoc analyses showed that MCL3 had significantly more visits than MCL1 (p = 0.013) and MCL2 (p = 0.031) and there was no difference between MCL2 and MCL3 (p = 0.397). (**B**) Encounters/Visits type per Mclust subgroup. Percent of visits/encounters with medical providers and/or services did not differ between Mclust subgroups (χ^2^ = 0.220, p = 1). All three groups used comparable proportion of services provided throughout their treatment; the most used services were physical rehabilitation and chiropractor visits. *ACU* acupuncture, *ALT* alternative medicine, *BEH* behavioral health, *CHIR* chiropractic, *MED* medical, *NAT* naturopathic, *NRS* nurse, *PR* physical reconditioning, *VAR* variable.

We then examined the degree to which our baseline cluster analysis of patient reported outcomes impacted recourse utilization in the transdisciplinary pain program. One-way ANCOVA with Mclust subgroup as a fixed factor, sex and age as covariates showed that groups differed significantly in the total number of days spent in the pain rehabilitation program (F(2,1,1) = 3.72, p = 0.023) and the group difference approached significance in the number of visits/encounters (F(2,1,1) = 2.823, p = 0.060) (Fig. 3a). Post-hoc analyses showed that the total number of days spent in the program was significantly higher in MCL3 compared to MCL1 (p = 0.039). This difference approached significant between MCL1 and MCL2 (p = 0.065) and there was no significant difference between MCL2 and MCL3 (p = 0.324). Likewise, MCL3 had significantly more visits than MCL1 (p = 0.013) and MCL2 (p = 0.031) and there was no difference between MCL2 and MCL3 (p = 0.397). Examination of type of visits/ encounters with medical providers and/or services did not differ between the subgroups (χ^2^ = 0.220, p = 1). In other words, all three subgroups used comparable proportion of services provided throughout their treatment (Fig. 3b). The most used services were physical rehabilitation and chiropractor visits.

### Cluster characterization: treatment trajectories

**Figure 4:**
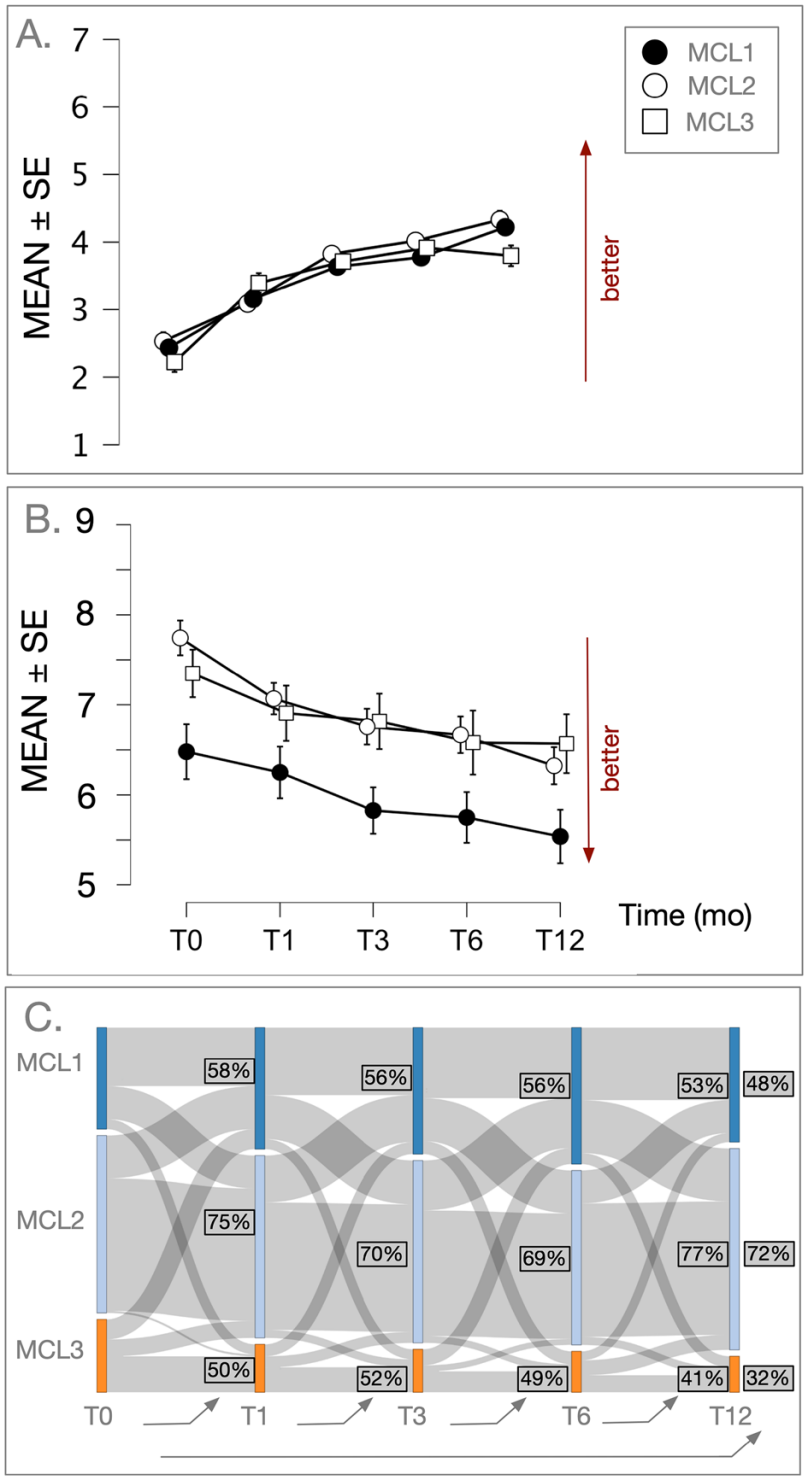
(**A**) Patient Global Impression of change over time (Mean ± SE) in Mclust subgroup completers (n = 665). PGIC showed overall increase indicating improvement over the course of treatment in all three Mclust subgroups, which was significant (F(4,8,4,4) = 4.128, p = 0.002). When Mclust subgroups were examined at each time point, significant subgroup effect was observed at six months (Time6) (F(2,1,1) = 3.366, p = 0.035) and approached significance at the last time point (Time12) (F(2,1,1) = 2.639, p = 0.072). Post-hoc comparisons showed that MCL2 had significantly lower PGIC scores than MCL1 at 6 months (p < 0.05) but not at 12 months (p > 0.05). Conversely, at 12 months, MCL3 had significantly lower PGIC scores than MCL1 (p < 0.05), a difference that approached significance compared to MCL2 group (p = 0.086). (**B**) PEG over time (Mean ± SE) in Mclust subgroups completers (n = 675). Examination of PEG scores over the course of treatment showed overall decline suggesting improvement in pain symptoms in all there subgroups (F(4) = 27.275, p < 0.001)***.*** When subgroup effects were examined at each time point, PEG scores were consistently and significantly lower in MCL1 compared to MCL2 and MCL3 (p’s < 0.05), and there was no significant difference between MCL2 and MCL3 (p’s > 0.05). c.f. text for further details. (**C**) Cluster dynamics (n = 353). Sankey plot indicating the transition of patients across clusters over the five longitudinal time points. Width of lines reflect the extent of movement between time points. % of patients staying and moving out of clusters are shown.

Repeated measures ANOVA was used to examine the effects of time (or assessment point) on Patient’s Global Impression of Change (PGIC) with sex and age as covariates. PGIC at each time point showed overall increase indicating improvement over the course of treatment in all three groups, which was significant (F(4,8,4,4) = 4.128, p = 0.002) (Fig. 4a). All timepoints were significantly different (p’s < 0.001) except the difference in PGIC scores between t3 (3 month) and t6 (6 months) (p = 0.145). Since the between subgroup difference in PGIC at each time point was of interest, we used one-way ACNOVAs (covariates: age, sex) to examine this effect. Significant subgroup effect was observed at six months (Time6) (F(2,1,1) = 3.366, p = 0.035) and approached significance at the last time point (Time12) (F(2,1,1) = 2.639, p = 0.072). Post-hoc comparisons (Holm corrected) showed that MCL2 had significantly lower PGIC scores than MCL1 at 6 months (p < 0.05) but not at 12 months (p > 0.05). Conversely, at 12 months, MCL3 had significantly lower PGIC scores than MCL1 (p < 0.05), a difference that approached significance compared to MCL2 group (p = 0.086) (Fig. 4a). Examination of PEG scores over the course of treatment showed overall decline suggesting improvement in pain symptoms in all there subgroups (F(4) = 27.275, p < 0.001) (Fig. 4b). Changes in PEG scores between the evaluated timepoints were significant (p’s < 0.05), except for t3 to t6. Subgroup effects at each time point were examined with one-way ANCOVAs. As mentioned above, PEG was significantly lower at baseline in MCL1 compared to MCL2 and MCL3, and there was no significant difference between MCL2 and MCL3. This between subgroup difference remained the same and significant throughout the course of treatment (p’s < 0.05).

### Cluster characterization: cluster dynamics

Transition of patients across clusters over the five longitudinal time points are shown in Fig. 4c. We found that ~ 70% of patients with MCL2 remained in the same cluster over the course of treatment. Conversely, ~ 50% of patients with MCL1 and MCL3 changed cluster assigned as treatment progressed. While group membership as defined through predictions from the baseline cluster model was transient, the group fit retained a high degree of coherence (cosine similarity ~ 0.91 and chi-square p < 1 × 10^–15^).

## Discussion

The goal of this work was to address the knowledge gap in the field whether a personalized medicine approach, such as transdisciplinary pain care, can successfully adapt to the needs of a heterogeneous chronic pain population. To address this gap, the unsupervised, data-driven modeling was used to identify whether subgroups of patients with chronic pain undergoing transdisciplinary treatment: (1) can be derived based upon self-reported outcome measures at baseline (or before treatment initiation), (2) are clinically validated based on their clinical diagnosis and medication use, and differ in (3) resource utilization and (4) treatment trajectories over 1-year of transdisciplinary treatment. Several important findings were observed. First, we identified three robust clusters based on baseline patient reported outcomes. Second, our data-driven subgroups showed a pattern of significant differences across clinical diagnosis and presentation. Third, we found that all three subgroups improved over time suggesting that treatment could be considered effective across the numerous presentations of chronic pain patients; this is consistent with the goals of transdisciplinary approach to chronic pain care^11^. Nevertheless, differences in resource utilization and treatment prognosis between the subgroups were noted that we discuss below.

Several studies used cluster-based approach in chronic pain patients and are consistent with our results, in that generally, the majority of these studies identified 2–4 clusters based on pain and mental health symptom severity^4,7–9,12–14^. A Spanish study in adult population (n = 1957) identified two clusters separable by the severity of pain and mental health problems^8^. Likewise, a Swedish study in older adults with chronic pain (n = 2415), revealed four clusters^9^, similar to another Swedish study of patients with chronic pain (n = 4665) based on data collected from the Swedish quality registry^7^. A German study of patients with low back pain (n = 1238) in primary care identified four clusters^13^, while a Canadian study in veterans with chronic pain (n = 2754) found three clusters^14^. An older U.S. study of patients with chronic pain (n = 453) in treatment at a pain center used data from Symptom Checklist 90(SCL-90) and a comprehensive pain evaluation questionnaire and found three clusters^12^. A cluster analysis of a larger number(n = 15,480) of members of a U.S. health maintenance organization with two or more chronic conditions identified ten clusters, one of which was dominated by patients with chronic pain, a majority of whom also had mental health conditions, and about half had obesity^15^. A recent large scale. (n = 11,448) U.S. study used only pain-agnostic symptoms and identified a three clusters solution based on graded symptom severity^4^; negative-affect-related symptoms were the key determinants in cluster assignment in this large study^4^. This work also examined longitudinal timecourse from a subset of patients (n = 1283) who completed a follow-up survey between 3 and 12 months after the initial assessment and found that the severity of symptoms at follow-up could be reliably predicted by baseline subgrouping^4^. These prior studies confirm that cluster analysis can consistently identify unique and potentially stable subgroups of people with chronic pain irrespective of chronic pain population. Our findings are consistent with these studies in symptom distribution and unique cluster identification among chronic pain sufferers thus providing the premise of our treatment trajectories through transdisciplinary pain care being conceptually generalizable to other population clusters.

Our unsupervised, data-driven approach identified three stable clusters of patients with chronic pain based on nine patient-reported pain and psychiatric symptoms. We found that all three groups reported moderate-severe chronic pain at baseline. Likewise, the proportion of patients on opioid medication was not significantly different between the three subgroups (see below for further discussion). These results suggest that pain symptoms reported by the patients at baseline were unlikely to drive the cluster separation in our study. This is consistent and extends recent findings on the role of pain intensity measure in determining reliable cluster solutions^4^. However, despite similar clinical pain presentation in all three subgroups, several striking differences were observed. We found that group 1, or MCL1, was characterized by lower ratings on all but substance use measures at baseline. Subjective ratings and clinical presentation of this group is consistent with the least impaired chronic pain population, in terms of pain and pain-related disability. We observed similar findings in our recent brain imaging study whereby individuals with low psychological and pain measures clustered out based on the pattern of brain connectivity at rest^16^. We found that the brain connectivity at rest in this low symptom group was consistent with resilient brain function, suggesting that even though these individuals have chronic pain and/or trauma, they have the capacity to deal with their symptoms in a more resilient way and thus report lower symptoms^16^. Nevertheless, our least impaired chronic pain population (MCL1) did not show the lowest scores on substance use measure; it is possible that these individuals are likely to self-medicate and thus report lower ratings. We found that group 2, or MCL2, was characterized by the highest disability due to back pain. Compared to the least impaired group, this group showed less improvement at 6 months but similar improvement at 12 months follow-up. Otherwise, this group was clinically and psychologically very similar to the least impaired chronic pain group, or MCL1. This group was also slightly younger and, similarly to MCL1, had a greater proportion of women consistent with the sexual dimorphism in chronic pain prevalence in the general population^17^. We found that group 3, MCL3 reported more mental health symptoms (depression, anxiety, alcohol and substance use), had the greatest proportion of major depressive disorder diagnosis, were prescribed higher dose of daily opioids, stayed in the program longer and showed less improvement at the end of the follow-up, or at 12 months post program initiation. This group is consistent with the most impaired, or high-impact, chronic pain group with more severe pain, multiple mental health concerns and showed similar prevalence to that in the U.S. adult population (~ 16% in our study)^18,19^. Higher dose of daily opioids found in this group are consistent with our recent work that showed association of daily opioids with mental health status rather than pain severity^20^. Likewise, poorer improvement overtime and higher disability in this high-impact pain group is consistent with the literature^21^. Particularly for those also affected by psychiatric disorders, chronic pain is an important driver of health care utilization in primary and other health care services^22^. Those whose chronic pain is aggravated by co-occurring mental health conditions show poorer treatment outcomes, increased pain severity and disability^23^. Thus, our results provide clinical validation for the identified subgroups within a large sample of chronic pain patients. It is also interesting, that unlike the other two clusters, this high impairment, high-risk subgroup had greater proportion of men.

Importantly, we found that overall, all three groups improved comparably overtime. We found that patient’s global impression of change (PGIC) increased as treatment progressed, indicating improvement. Likewise, subjective pain rating (PEG) decreased as treatment progressed, indicating gradual decrease in perceived pain impact over time. From this perspective, treatment was effective. This finding was not surprising, considering the transdisciplinary nature of the program whose goals are to maximize patient’s benefit^11^. We believe that it is largely due to the fact that current treatment program is dictated by patient choice, which is guided by patient’s expectations and is adapted accordingly^24^. In other words, treatment plan is modified according to patient’s choices, which are based on the most positive expectation of clinical benefit thereby potentially incurring greatest benefit^24^. It is plausible that a less adaptive treatment plan would show greater divergence of outcomes across the subgroups. Nevertheless, the high-impact chronic pain group (i.e., MCL3) reached ceiling effect around 6 months. This points to the possibility that high-impact chronic pain patients may need more guidance and/or more aggressive approaches in their treatment. Perhaps targeting mental health prior to targeting pain issues would mitigate these ceiling effects. It is generally accepted that non-volitional, or not controlling their interventions, has poorer outcomes compared to volitional treatment choices^25^. Likewise, individual vs. group approach should be considered when high-risk patients are involved^26^. Interestingly, despite symptoms improvement over time, clusters were relatively stable, in that the majority of patients remained with their own cluster. This was mostly pronounced in MCL2, or the younger group with the highest back-pain related disability, suggesting that patients within this group have unique features that may not change with treatment despite symptoms improvement.

A key aspect of the current work is translatability to clinical practice. Specifically, this work presents insight as to the types of patients that approach transdisciplinary treatment and provides insight into how this presentation may affect the course and trajectory of treatment. Importantly, it should be noted that there were striking similarities between treatment paths in the transdisciplinary clinic across a variety of patient types. This bolsters the trend that a transdisciplinary approach is becoming a “go-to” method for chronic non-cancer pain^10^. However, there are potentially warning signals that some subject populations may tend to have a waning benefit after a year of treatment, thus potentially encouraging a re-visitation of treatment needs and goals at the 6-month timepoint in this population.

Several limitations should be noted. First, the generalizability of this data needs further study. The current sample included patients from a regionally homogeneous population (San Bernardino and Riverside Counties, CA). However, the findings in this sample appear to replicate similar work done in other samples. Secondly, there are numerous cluster-based or unsupervised modeling approaches that could be taken, which potentially, may affect the resulting solution. We selected the *Mclust* approach as this was similar to the approaches taken in prior work and thus added more directly to this body of literature. Similarly, the determination of the ultimate number of clusters was limited to the range of prior samples, thus enhancing replication and contrast to prior work (see Supplementary Material for other clustering solutions). Thirdly, the variables that were selected for this model may have impacted the resulting groups^4^. The sample represented the measures that were selected initially for maximizing clinical utility and reduced down to reflect those measures that were deemed most informative to potential pain phenotype grouping. This culling of measures was important to better reduce the influence of non-critical measures that could allow the model to overfit to non-critical features in the sample. Finally, the measures used for cluster formation did not include important biological and genetic measures that have been deemed important in understanding pain^27^. However, the use of easily administered survey items greatly enhances the portability and clinical utility of the current findings.

In summary, we used unsupervised, data-driven modeling approach to examine whether patients with chronic pain undergoing transdisciplinary pain care at the same clinic could be separated based on patient reported outcomes that are known to affect the expression of chronic pain. We found that, even in an adaptive transdisciplinary treatment program, subgroups with higher pain and psychiatric symptoms exist, may need more care, and may have greater treatment resistance detectable in long term care.

## Methods

### Study design and procedures

Study was approved by the Western Institutional Review Board. Data collection was performed in accordance with relevant guidelines and regulations. All patients included in this study provided informed consent, received transdisciplinary pain treatment at Desert Clinic Pain Institute and Summit Institute (now Savas Health https://savashealth.com/) and completed a set of standardized assessment tools (see below) at baseline/screening visit month 0 (t0), month 1 (t1), month 3 (t3), month 6 (t6) and month 12 (t12). The overview of the program is outlined in Supplemental Material 1 and the study design is summarized in Fig. 1. Briefly, there is a total of four departments, as well as combined educational classes working together to establish these goals (Medical Department, Behavioral Department, Physical Reconditioning Department, and Department of Alternative Care). There are total of three phases. Phase 1 (~ 1 month “Rescue”) is composed of weekly visits (weeks 1–4, total of no less than 10 combined encounters/visits) with a goal of stabilize, engage, explain and treat. Phase 2 (~ 5 months “Restore”) is composed of twice/month visits (months 2–6, total of no less than 12 combined visits/encounters), while Phase 3 (~ 6 months, months 7–12, “Re-entry”) is composed of twice per month visits (total of no less than 12 combined visits/encounters and at least one visit with the Medical Department per month).

Data were analyzed retrospectively and cover the time from 03/01/2016 to 05/31/2019.

### Subjects

A total of 3296 patients with chronic pain lasting more than 3 months undergoing clinical care at the Desert Clinic Pain Institute and Summit Institute completed the self-reported outcomes battery at baseline and were used for current analysis. Patients were included if they were insured by the Inland Empire Health Plane (IEHP – Medicare and Medicaid plan) and were ≥ 18 years of age and met at least one of the following inclusion criteria and none of the exclusion criteria (mandatory to be included in the program): (1) Current high dose Opioid use (Dose at or above 120 mg MED per day); (2) Help with tapering/discontinuing of medication needed; (3) Presents with psychiatric illness or symptoms complicating treatment of chronic pain; (4) Chronic pain refractory to usual interventions; (5) Member's work or lifestyle has been significantly impaired due to chronic pain; (6) Member not satisfied with current pain care outcomes; 7) Complex pain conditions. Patients were excluded if: (1) Is not covered by Inland Empire Health Plan (see Supplement for detailed description of the program).

### Assessment battery

Subjects completed the following self-reported battery of questionnaires: (1) The Numeric Pain Rating Scale (NPRS; 0- 10 Likert scale) corresponding to current pain experienced^28^; (2) Pain Intensity and Interference (PEG)^29^; (3) Oswestry Low Back Pain Disability Questionnaire (ODQ), which is considered the ‘gold standard’ of low back functional outcome tools^30^; (4) Pain Disability Index (PDI)^31^ to measure overall pain-related disability; (5) Pain Catastrophizing Scale (PCS) to measure individual’s catastrophic thinking about their pain^32^; (6) Patient Health Questionnaire-9 (PHQ-9) to evaluate depressive symptoms^33^; (7) General Anxiety Disorder (GAD-7) to measured anxiety symptoms^34^; (8) DAST-10 Questionnaire (DAST) to assess drug use, not including alcohol or tobacco use, in the past 12 months^35^; (9) The Alcohol Use Disorders Identification Test (AUDIT) to assess alcohol consumption, drinking behaviors, and alcohol- related problems^36^; and (10) Patient’s Global Impression of Change (PGIC), a 7 point Likert scale rating the subjects change in activity limitations, symptoms, emotions and overall quality of life since beginning of the treatment; this measure was used to evaluate longitudinal outcomes of treatment. Note, for the baseline rating, patients were asked to rate relative to all past treatments tried. For the following time-points, the rating referred to the beginning of the current treatment. The following anchors were used: 1 = No change (of condition has got worse); 2 = Almost the same, hardly any change at all; 3 = A little better, but no noticeable change; 4 = Somewhat better, but the change has not made any real difference; 5 = Moderately better, and a slight but noticeable change; 6 = Better, and a definite improvement that has made a real and worthwhile difference; 7 = A great deal better, and a considerable improvement that has made all the difference.

### Data cleaning and cluster size determination

All statistical analyses were performed using the statistical program R version 4.0.2, RStudio. The missing values in the self-report measures were imputed using multivariate imputation by chained equations (MICE) consisting of 5 multiple imputations with 3 iterations, in R using the MICE package version 3.12.0. For the self-report measures, imputation was used for patients that had < 20% of a questionnaire missing. Patients were excluded if they had > 20% of the questionnaire missing. Ultimate cluster size was determined using two approaches. Clusters were selected from a range of groups from 1 to 6 based on prior literature^4,7–9,12–14^. The quality of a given cluster was assessed based on the Bayesian Information Criterion (BIC) score for the entire sample (R package: mclust, version 6.0.0), as well as well as the Jaccard index of overlap between subsamples of the data that were modified through bootstrapping, jittering, and replacement by noise (R package: fpc, version 2.2-9). From this selection similar fits were achieved with 3, 4, and 6 cluster solutions (depending on metric). The three-group solution was selected due to maximal parsimony, and, if successful, optimal clinical efficiency.

### Cluster discovery

An unsupervised subgroup classification was performed using subgrouping within *Mclust* in *R* (version 6.0.0)^37^ in order to identify whether clinically relevant subgroups of subjects (1) can be derived based upon self-reported outcome measures at baseline, (2) are clinically validated and (3) differ in treatment trajectories over the 1 year of follow-up treatment. *Mclust* is a contributed R package for model-based clustering, classification, and density estimation based on finite normal mixture modelling. It provides functions for parameter estimation via the mixture estimation (EM) algorithm for normal mixture models with a variety of covariance structures, and functions for simulation from these models. Also included are functions that combine model-based hierarchical clustering, EM for mixture estimation and the BIC in comprehensive strategies for clustering, density estimation and discriminant analysis. For trajectories analysis, PGIC and PEG scores were examined at each time point, i.e., baseline (t0), 1 month (t1), 3 months (t3), months (t6) and 12 months (t12). To assess cluster dynamic, i.e. to examine patient movement across clusters between the baseline and subsequent time points we applied the cluster modeling developed at baseline at each following point (mclust::predict.Mclust). This confirmatory analysis was applied only to patients that had complete data at each time point (n = 353). The transitions were depicted across time using a Sankey diagram (R: networkD3 version 0.4).

### Cluster characterization analysis

Statistical analysis of clinical and psychological variables was conducted in R and JASP (JASP Team (2020). JASP (Version 0.14.1) (Computer software). ANCOVAs, *t*-tests and chi-square tests were used to compare Mclust subgroups on clinical and demographic variables (i.e., age). Results were corrected using Holm method. For the treatment trajectory’s analysis, we used repeated measures ANOVA with Mclust as a fixed factor and assessment time (t0, t1, t3, t6, t12) as the repeated measure to examine effects of time. Groups were compared at each time point using ANCOVAs. Sex and age were used as covariates in all of the analyses.

## Supplementary Information

Supplementary Information.

## Data Availability

The datasets used and/or analyzed during the current study available from the corresponding author on reasonable request.
