# Supplementary material for: Unsupervised learning for prognostic validity in patients with chronic pain in transdisciplinary pain care
Source: Sci Rep. 2023 May 10;13:7581. doi: 10.1038/s41598-023-34611-z (PMC10172363; doi:10.1038/s41598-023-34611-z)
Supplement: Supplementary file 1 — Supplementary Information. [file 41598_2023_34611_MOESM1_ESM.pdf]

Supplemental Material 1:

Desert Clinic Pain Institute/Summit Institute (now Savas Health) Phase-based transdisciplinary treatment system for complex chronic pain.

Inclusion Criteria:

To be included into the program, patients needed to be members of the Inland Empire Health Plan (IEHP).

Additional inclusion Criteria:

Patient with chronic pain (more than 3 month in duration) and at least one of the following:

1. Current high dose opioid use (Dose at or above 120 mg MED per day)
2. Help with tapering/discontinuing of medication needed
3. Presents with psychiatric illness or symptoms complicating treatment of chronic pain
4. Chronic pain refractory to usual interventions
5. Member's work or lifestyle has been significantly impaired due to chronic pain
6. Member not satisfied with current pain care outcomes
7. Complex pain conditions

Program Organization:

The treatment program is one (1) year long.

The program consists of three (3) phases (Phase I "Rescue": month 1, Phase II "Restore": months 2-6, Phase III "Re-entry": months 7-12).

There are a total of 4 departments including the Medical Department, the Behavioral Department, the Physical Reconditioning Department, and the Educational and Alternative Treatment Department.

Four (4) departments:

- Medical Department
    - Medical Doctors, Nurse Practitioners, Physician Assistants, Interventional Pain Specialists
- Behavioral Department
    - Psychiatrists, Psychologists, Cognitive Behavioral Specialists
- Physical Reconditioning Department
    - Chiropractors, Physical Therapists, Fitness Instructors, Yoga & Tai Chi Masters
- Department of Alternative Care
    - Naturopathic Doctors, Acupuncturists, Dieticians

Program Schedule:

Weeks 1-4 of Month 1 (with a total of no less than 10 combined visits/encounters)

- Medical Department: 1-2 Visits/Encounters
- Behavioral Department: 4-8 Visits/Encounters
- Physical Reconditioning Department: 4-12 Visits/Encounters
- Education and Department of Alternative Care: 1-4 Visits/Encounters

Weeks 5-26 or Month 2-6 (with a total of no less than 12 combined visits/encounters)

- Medical Department: 5-10 Visits/Encounters
- Behavioral Department: 5-10 Visits/Encounters
- Physical Reconditioning Department: 1-10 Visits/Encounters
- Education and Department of Alternative Care: 1-12 Visits/Encounters

Weeks 27-52 or Month 7-12 (with a total of no less than 12 combined visits/encounters and at least one visit with the Medical Department per month)

- Medical Department:
    - 6-12 Medical Visits/Encounters
    - 0-4 Injections
- Behavioral Department:
    - 0-20 Individual Psychotherapy Visits/Encounters
    - 0-4 Group Psychotherapy Visits/Encounters
- Physical Reconditioning Department:
    - 0-72 Individual Visits/Encounters (Chiropractic, Functional Rehab, Movement Therapy, Yoga, Tai Chi, etc.)
    - 0-72 Group Therapy Visits/Encounters
- Education and Department of Alternative Care: 1-12 Visits/Encounters
    - 0-25 Weekly Lectures
    - 0-14 Alternative Treatments (Neuropathic, Massage, Acupuncture, Dietician, etc.)
- Department of Alternative Care:
    - 0-48 Visits/Encounters (Neuropathic Treatment, Massage, Acupuncture, Dietician, etc.)

Clustering Solutions

The number of clusters was chosen based on published literature in chronic pain patients (see Table below), optimal clinical efficiency and multiple iterations using several clustering approaches.

Study	Number of Patients	Number of clusters
(Jamison, Rock et al. 1988)	453	3
(Hirsch, Strauch et al. 2014),	1,238	4
(Duenas, Salazar et al. 2015)	1,957	2
(Larsson, Gerdle et al. 2017),	2,415	4
(Backryd, Persson et al. 2018)	4,665	4
(Reyes Velez, Thompson et al. 2021)	2,754	3
(Gilam, Cramer et al. 2021)	11,448	3

We have tested numerous clustering solution methodologies and limit testing in the determination of the optimal clustering. We found that both Kmeans and MClust provide plausible solutions (see below). The appropriate range for clusters for Kmeans appears to be between 1 and 6 clusters. The range of appropriate clusters for MClust appears to be between 2 and 4 clusters. Nbclust (which models a sample using a plethora of clustering of models for a voting) suggested 2 clusters (n=8), 3 or 6 clusters (n=3), and 4, 7, 9, or 15 clusters (n=2). As noted in the literature in the Table above the optimal solution in other literature appear centered on 3 or 4 clusters. As the collective set of information seems to suggest 3 or 4 clusters, we settled upon 3 for optimal parsimony.

G 1 to 7 mclust:

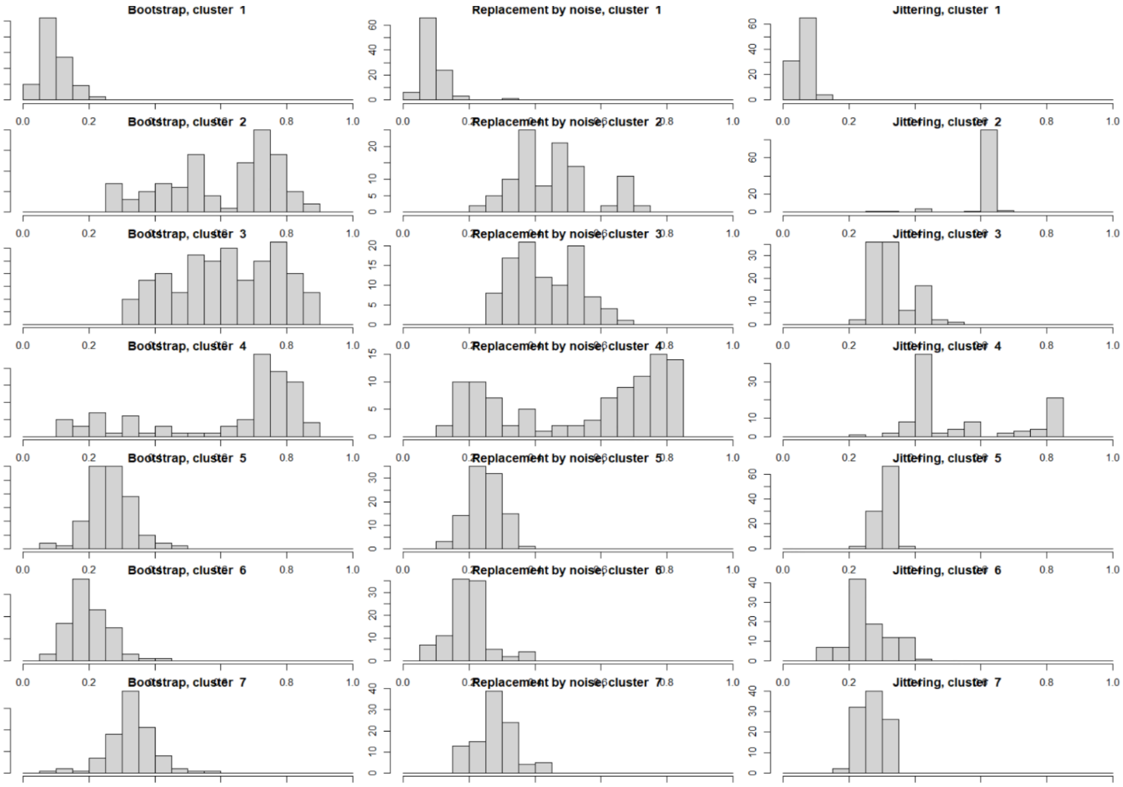

Kmeans:

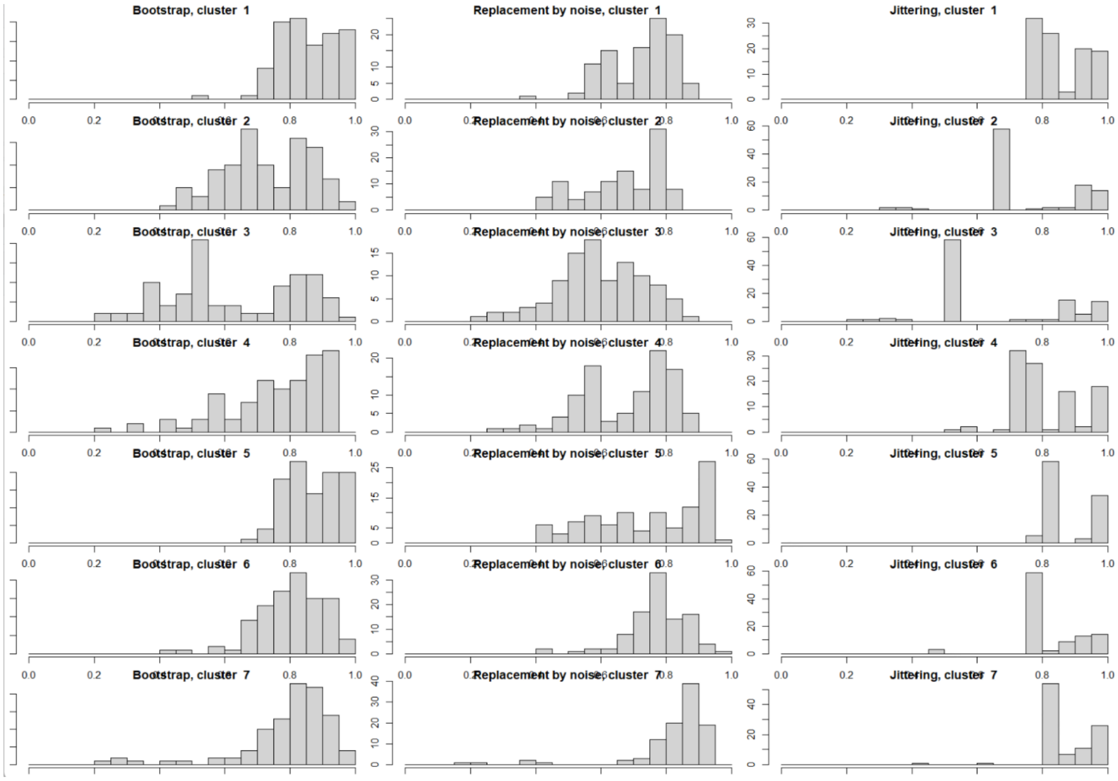

Hierarchical Clustering:

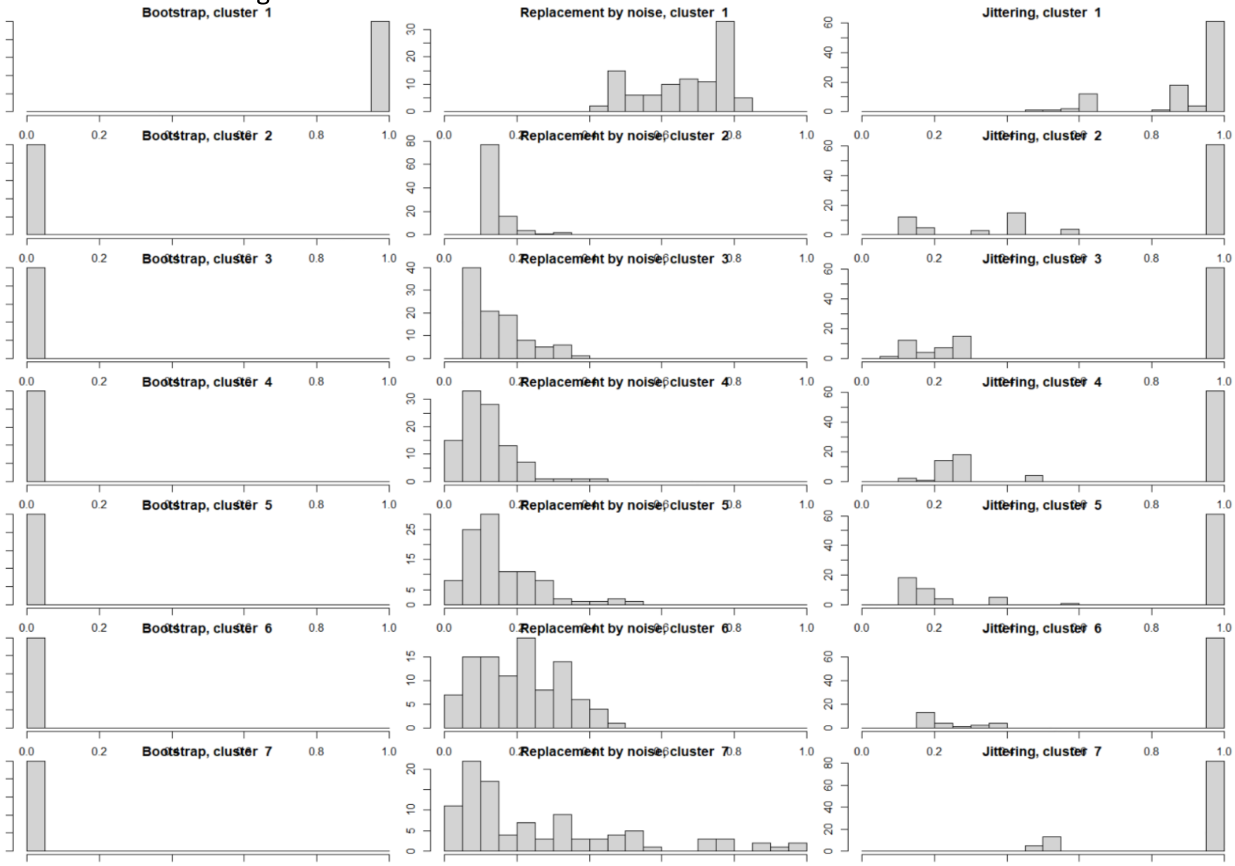

Graph-based (fast greedy):

Cluster	Sample Size
**1**	1703
**2**	92
**3**	1470
**4**	7
**5**	2
**6 to 27**	1

Graph-based (walk trap):

Cluster	Sample Size
**1 to 5**	2
**6 to 3291**	1

Supplementary Figure 1: Proportion of subjects endorsing PGIC rating in each cluster

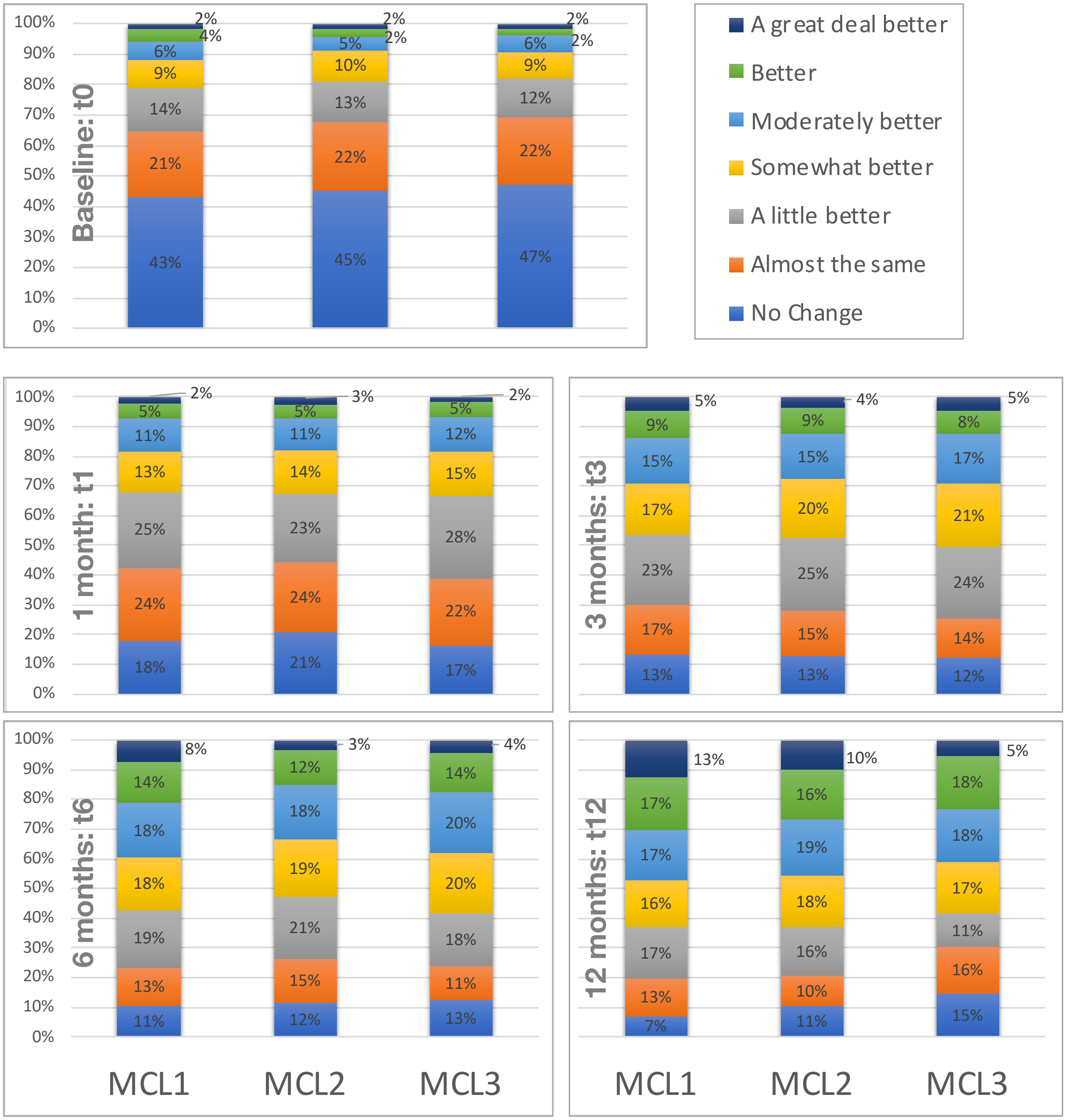

References

Backryd, E., E. B. Persson, A. I. Larsson, M. R. Fischer and B. Gerdle (2018). "Chronic pain patients can be classified into four groups: Clustering-based discriminant analysis of psychometric data from 4665 patients referred to a multidisciplinary pain centre (a SQRP study)." PLoS One **13**(2): e0192623.

Duenas, M., A. Salazar, B. Ojeda, F. Fernandez-Palacin, J. A. Mico, L. M. Torres and I. Failde (2015). "A nationwide study of chronic pain prevalence in the general spanish population: identifying clinical subgroups through cluster analysis." Pain Med **16**(4): 811-822.

Gilam, G., E. M. Cramer, K. A. Webber, 2nd, M. S. Ziadni, M. C. Kao and S. C. Mackey (2021). "Classifying chronic pain using multidimensional pain-agnostic symptom assessments and clustering analysis." Sci Adv **7**(37): eabj0320.

Hirsch, O., K. Strauch, H. Held, M. Redaelli, J. F. Chenot, C. Leonhardt, S. Keller, E. Baum, M. Pfingsten, J. Hildebrandt, H. D. Basler, M. M. Kochen, N. Donner-Banzhoff and A. Becker (2014). "Low back pain patient subgroups in primary care: pain characteristics, psychosocial determinants, and health care utilization." Clin J Pain **30**(12): 1023-1032.

Jamison, R. N., D. L. Rock and W. C. Parris (1988). "Empirically derived Symptom Checklist 90 subgroups of chronic pain patients: a cluster analysis." J Behav Med **11**(2): 147-158.

Larsson, B., B. Gerdle, L. Bernfort, L. A. Levin and E. Dragioti (2017). "Distinctive subgroups derived by cluster analysis based on pain and psychological symptoms in Swedish older adults with chronic pain - a population study (PainS65+)." BMC Geriatr **17**(1): 200.

Reyes Velez, J., J. M. Thompson, J. Sweet, J. W. Busse and L. VanTil (2021). "Cluster analysis of Canadian Armed Forces veterans living with chronic pain: Life After Service Studies 2016." Can J Pain **5**(1): 81-95.
